# Assessing the quality of interdisciplinary rounds

**DOI:** 10.1186/cc12459

**Published:** 2013-03-19

**Authors:** EC Ten Have, JE Tulleken

**Affiliations:** 1 Universitair Medical Center Groningen, the Netherlands

## Introduction

Interdisciplinary rounds (IDRs) in the ICU are increasingly recommended to support quality improvement and to reduce conflicts, but uncertainty exists about assessing the quality of IDRs. We developed, tested, and applied a scoring instrument to assess the quality of IDRs in ICUs.

## Methods

A literature search was performed to identify criteria for instruments about assessing team processes in the ICU. Then, 10 videotaped patient presentations led by different intensivists were analyzed by Delphi rounds. Appropriate and inappropriate behaviors were highlighted. The IDR-Assessment Scale was developed and statistically tested. The inter-rater reliability was evaluated by rating nine randomly selected videotaped patient presentations by three raters. Finally, the scale was applied to 98 videotaped patient presentations during 22 IDRs in three ICUs for adults in two hospitals in Groningen.

## Results

The IDR-Assessment Scale had 19 quality indicators, subdivided into two domains: Patient Plan of Care, and Process. The domain Patient Plan of Care reflects the technical performance from the initial identification of a goal to the evaluative phase. The domain Process reflects the team processes that are important to ensure that the appropriate plan of care is agreed, understood, and executed as planned by all care providers. Indicators were essential or supportive. The inter-rater reliability of nine videotaped patient presentations among three raters was satisfactory (κ = 0.85). The overall item score correlations between three raters were excellent (*r *= 0.80 to 0.94). Internal consistency in 98 videotaped patient presentations was acceptable (a = 0.78). Application to 22 IDRs led by 14 different intensivists in three ICUs in two hospitals demonstrated that indicators could be unambiguously rated. The staff and management of all three ICUs that were rated had considered their IDRs to be adequately performed, and they were surprised by these study results.

## Conclusion

This study showed that the quality of IDRs can be reliably assessed for patient plan of care and process. The IDR-Assessment Scale had satisfactory inter-rater reliability, excellent overall item score correlations, and acceptable internal consistency. Our instrument may provide feedback for ICU professionals and managers to develop adjustments in quality of care. Testing the IDR-Assessment Scale in other ICUs may be required to establish general applicability.
